# Apolipoprotein E polymorphism and the risk of aneurysmal subarachnoid hemorrhage in a South Indian population

**DOI:** 10.1186/s11658-017-0059-8

**Published:** 2017-11-29

**Authors:** Arati Suvatha, Sibin Madathan Kandi, Dhananjaya Ishwara Bhat, Narasinga Rao, Vikas Vazhayil, Chetan Ghati Kasturirangan

**Affiliations:** 1 Department of Human Genetics, National Institute of Mental Health and Neuro Sciences, Bangalore, Karnataka 560029 India; 2 Department of Neurosurgery, National Institute of Mental Health and Neuro Sciences, Bangalore, 560029 India

**Keywords:** Aneurysmal subarachnoid hemorrhage, Apolipoprotein E, Polymorphism, Posterior communicating artery aneurysm

## Abstract

**Background:**

The rupture of a brain aneurysm causes bleeding in the subarachnoid space. This is known as aneurysmal subarachnoid hemorrhage (aSAH). We evaluated the association of apolipoprotein E (*APOE*) polymorphism and the risk of aSAH in a South Indian population.

**Methods:**

The study was performed on 200 subjects with aSAH and 253 healthy control subjects. Blood samples (5 ml) were used to isolate DNA and genotyping was performed for rs7412 and rs429358 using a Taqman allelic discrimination assay. Statistical software R.3.0.11 was used to statistically analyze the data and a *p* value < 0.05 was considered as statistically significant.

**Results:**

We found a significant association with the risk of aSAH in ε3/ ε4 genetic model (OR = 1.91, 95% CI = 1.16–3.14, *p* = 0.01). However, in the other genetic models and allele frequency, there was no significant association with the risk of aSAH. In subtyping, we found a significant association of ε2 allele frequency with posterior communicating artery (PCOM) aneurysm (OR = 3.59, 95% CI = 1.11–11.64, *p* = 0.03).

**Conclusion:**

Our results suggest that *APOE* polymorphism has an influence on the risk of aSAH in this South Indian population, specifically in the PCOM subtype.

## Introduction

Aneurysmal subarachnoid hemorrhage (aSAH) is a life-threatening condition that accounts for 5% of strokes [1]. aSAH occurs due to the rupture of an aneurysm that is commonly found in the Circle of Willis region in the brain [2]. A recent report suggested that the overall incidence of aSAH was approximately 9 in 100,000 people per year, but that this can vary significantly with geographical region [3]. In India, the incidence rate was determined as 21.8 in 100,000 people per year [4]. Japan and Finland have higher incidences of aSAH – 22 and 19.7 in 100,000, respectively [5]. Lower incidence rates were reported for South and Central America [3].

Genetic and environmental risk factors play a key role in the formation and rupture of aneurysms. Genome-wide association studies of aSAH in different population have identified many new risk loci associated with aSAH [6].

Apolipoprotein E (*APOE*) is a 299-amino acid protein mainly synthesized by the liver and macrophages [7]. In human beings, it is located in the long arm of chromosome 19 and the protein is encoded by exon 4 [8]. In the brain, it is synthesized by astrocytes and helps in the transport of cholesterol to neurons [9]. *APOE* also helps in neuronal repair [10], cerebral glucose metabolism [11], and differentiation and migration of neurons [12]. The *APOE* gene is polymorphic with three different alleles (ε2, ε3, ε4) and six different genotypes [13]. The combination of polymorphisms of rs7412 and rs429358 will determine the six possible genotypes [14].

In the Asian population, the allele frequencies for ε2, ε3 and ε4 were 5%, 86% and 9%, respectively. The most common genotype in the Asian population is ε3/ε3 (74%), followed by ε3/ε4 (16%), ε2/ε4 (13%), ε2/ε3 (8.5%), ε4/ε4 (0.7%) and ε2/ε2 (0.1%) [15]. The frequencies of *APOE* alleles do not vary with gender [16]. The ε4 allele is associated with unfavorable outcomes after traumatic brain injury [17, 18] and aSAH in the Chinese and Italian population [19, 20]. We performed a meta-analysis that suggested that there is significant association with the risk of aSAH in ε2/ε2 vs. ε3/ε3, ε2/ ε3 vs. ε3/ε3 and ε2 vs. ε3 genetic models and ε2 allele frequency [21].

There is no study that has addressed the relationship between *APOE* polymorphism and the risk of developing aSAH in the Indian population. The aim of this study is to investigate the association of *APOE* polymorphism and aSAH in a South Indian population.

## Materials and methods

### Study population

The subjects were 200 patients with aneurysmal subarachnoid hemorrhage recruited from the Department of Neurosurgery, NIMHNAS, Bangalore, India, and 253 ethnicity-, age- and sex-matched healthy controls were selected randomly from the general population in 2015–2017. The healthy controls were unrelated to the patients. The patients and control groups were all from the Dravidian Kannada-speaking population of South India.

The inclusion criteria for patients with aSAH were diagnosis of aneurysmal SAH with the presence of symptoms suggestive of aSAH combined with subarachnoid blood found with the CT scan and a proven aneurysm found via conventional angiography. The exclusion criteria for selecting patients were: the presence of neuropsychiatric conditions like dementia, Parkinson’s disease, epilepsy and psychoses; or if SAH resulted from a mycotic aneurysm, arterio-venous malformation or trauma. Demographic and clinical details were collected directly from the patients using structured questionnaires and data from the medical records section.

The inclusion criteria for healthy controls were: the absence of aneurysm, checked with digital subtraction angiography; similar demographic characteristics to the patient group (age, sex, ethnicity and dietary habits); no medical history of hemorrhage and no family history of aSAH in first-degree relatives.

The study protocol was approved by the Institute of ethics committee for human studies, NIMHANS, Bangalore. Written informed consent was obtained from all the participants.

### DNA extraction and genotyping

Blood samples (5 ml) were collected from all the participants. Genomic DNA was isolated from the blood using the commercially available Machery-Nagel (MN) kit according to the manufacturer’s protocol. The purity and quantity of DNA was analyzed using a Nanodrop ND2000c spectrophotometer. DNA with a purity of 1.75–1.85 was used for genotyping analyses.

Genotyping of rs7412 and rs429358 was performed using a Taqman allelic discrimination assay (Applied Biosystems) with a commercially available primer probe set (assay ID C_904973_10, C_3084793_20). Genotyping was performed in duplicates using an Applied Biosystem7500 Fast Real-Time Cycler.

### Statistical analysis

R.3.0.11 statistical software was used to statistically analyze the data. The continuous variables were expressed as means ± SD and categorical variables as absolute values and percentages. The demographic characteristics of the patients and controls were compared using the χ2 test for all categorical variables. Differences in genotype and allele frequencies between groups were analyzed using the χ2 test. Association between *APOE* genotypes or alleles and aSAH risk were expressed as the odds ratio (OR) with 95% confidence intervals (CI), adjusted for the confounding effects of smoking, hypertension, drinking and diabetes mellitus using the logistic regression model. The Hardy–Weinberg equilibrium calculation was performed using an online tool at http://www.oege.org/software/hwe-mr-calc.shtml. *p* < 0.05 was considered statistically significant.

## Results

### Study population characteristics

The characteristics of patients with aSAH and healthy controls were shown in Table 1. Clinical data were available for all patients and healthy control subjects and there was no statistical significance in gender difference. The mean age was slightly higher in aSAH patients than in the controls with a *p* value of 0.170. Smoking and alcohol consumption were slightly more common in patients. The frequencies of hypertension and diabetes mellitus were also slightly higher in patients. The majority of the patients had aSAH with an ACOM (anterior communicating artery) aneurysm (43%) with a size of less than 15 mm (79.5%) and a WFNS grade of I (46%).

**Table 1:** Demographic and clinical characteristics of patients with aSAH and healthy controls

Characteristics	aSAH	Controls	*P*
Total no.	200	253	–
Sex (male/female)	77/123	114/139	0.160
Female (%)	61.50	69.50	–
Male (%)	38.50	57.00	–
Age (years)	50.72 ± 10.76	45.57 ± 17.73	0.170
Hypertension (yes/no)	109/91	105/148	0.006
Smoking (yes/no)	58/142	25/228	< 0.0001
Drinking (yes/no)	57/143	26/227	< 0.0001
Diabetes mellitus	71/129	60/193	0.005
Site of aneurysm (no./%)			
ACOM	86/43	–	
PCOM	12/6	–	
ICA	36/18	–	
MCA	37/18.5	–	
Multiple	22/11	–	
Basilar top	7/3.5	–	
Size of aneurysm (no./%)			
Small (< 15 mm)	159/79.5	–	
Large (15–25 mm)	37/18.5	–	
Giant (> 25 mm)	4/2	–	
WFNS Grade (no./%)			
Grade I	91/45.5	–	
Grade II	42/21	–	
Grade III	51/25.5	–	
Grade IV	16/8	–	

### *APOE* polymorphism and risk of aSAH

The distribution of the *APOE* genotype and allele frequencies are shown in Table 2. The distribution of genotype frequencies of the control group was in Hardy–Weinberg equilibrium (rs7412; *p* = 0.36, rs429358; *p* = 0.93). The *APOE* allele frequencies in the controls (*n* = 253; ε2 = 0.06; ε3 = 0.83; ε4 = 0.12) were similar to the data obtained from a study carried out by Das et al. in a South Indian population (*n* = 620; ε2 = 0.05; ε3 = 0.84; ε4 = 0.11; χ^2^ = 0.01; df = 2; *p* = 0.91) [22]. In our study, there was no significant difference in *APOE* genotypes (χ^2^ = 0.05; df = 5; *p* = 1) and allele frequencies (χ^2^ = 0.009; df = 2; *p* = 0.99) between the patients and the controls.

**Table 2:** Distribution of *APOE* genotypes and alleles in patients with aneurysmal SAH and in control subjects

	Genotypes						Allele frequency		
	ε2/ε2	ε2/ε3	ε2/ε4	ε3/ε3	ε3/ε4	ε4/ε4	ε2	ε3	ε4
Patients	2 (1.0)	18 (9)	1	134 (67)	45 (22.5)	0	23 (5.75)	331 (82.75)	46 (11.5)
Controls	4 (1.60)	15 (5.92)	5 (1.97)	194 (76.6)	34 (13.4)	1 (0.4)	28 (5.53)	437 (86.36)	41 (8.1)

Numbers in parentheses are percentages

A comparison of different genotype models of aSAH with healthy controls showed no significant difference in the data. The results of logistic regression analyses are shown in Table 3. A statistically significant association was found in the genotype model ε3/ε3 vs. ε3/ε4 (OR = 1.91, 95% CI = 1.16–3.14, *p* = 0.01). The comparisons of allele frequencies ε2 vs. ε3 (OR = 1.08, 95% CI = 0.61–1.91, *p* = 0.78), ε2 vs. ε4 (OR = 0.73, 95% CI = 0.36–1.46, *p* = 0.73) and ε4 vs. ε3 (OR = 0.67, 95% CI = 0.43–1.05, *p* = 0.0.08) did not show any statistical significance.

**Table 3:** Distribution of *APOE* genotypes and alleles in patients with aneurysmal SAH and control subjects

Genotypes	OR (95% CI)	P
ε2/ε2 vs. ε3/ε3	0.72 (0.13–4.01)	0.710
ε2/ε2 vs. ε3/ε4	2.64 (0.45–15.3)	0.270
ε3/ε3 vs. ε4/ε4	0.48 (0.01–11.92)	0.650
ε2/ε2 vs. ε2/ε3	2.4 (0.38–14.96)	0.340
ε2/ε2 vs. ε2/ε4	0.4 (0.02–6.17)	0.510
ε3/ε3 vs. ε2/ε3	1.73 (0.84–3.56)	0.130
ε3/ε3 vs. ε2/ε4	0.28 (0.03–2.5)	0.260
ε3/ε3 vs. ε3/ε4	1.91 (1.16–3.14)	0.01
ε4/ε4 vs. ε2/ε3	3.58 (0.13–94.31)	0.440
ε4/ε4 vs. ε2/ε4	0.81 (0.02–32.26)	0.910
ε4/ε4 vs. ε3/ε4	3.95 (0.15–100.13)	0.400
Alleles		
ε2 vs. ε3	1.08 (0.61–1.91)	0.780
ε2 vs. ε4	0.73 (0.36–1.46)	0.730
ε3 vs. ε4	0.67 (0.43–1.05)	0.080

When the aneurysms were classified according to location, size and WFNS grade and compared with different *APOE* genotype models and allele frequencies, only the PCOM aneurysm was statistically significant with ε2 vs. ε3 allele frequency (OR = 3.59, 95% CI = 1.11–11.64, *p* = 0.03). Classification of aneurysms according to *APOE* genotype frequency is shown in Table 4. Similarly, we performed comparisons between male vs. female, hypertensive vs. non-hypertensive, and diabetic vs. non-diabetic patients with different *APOE* genotype model and allele frequencies. None of the comparisons showed statistical significance with the *APOE* allele or genotype model.

**Table 4:** Distribution of *APOE* genotypes and allele frequencies according to aSAH subtypes

Variable	Case	ε 2/ ε2	P	ε 2/ ε3	P	ε 2/ ε4	P	ε 3/ ε3	P	ε 3/ ε4	P	ε 4/ ε4	P	ε 2 Vs ε3 (P)	ε 4 Vs ε3 (P)
Total	200	2		18		1		134		45		0			
Site of Aneurysm															
ACOM	86	0	0.62	7	0.86	1	0.69	57	0.89	21	0.78	0	0.69	0.61	0.57
PCOM	12	1	0.09	2	0.42	0	0.15	5	0.26	4	0.51	0	0.15	0.03	0.31
ICA	36	0	0.82	2	0.92	0	0.33	30	0.61	4	0.18	0	0.33	0.26	0.12
MCA	37	1	0.42	4	0.75	0	0.44	24	0.82	8	0.92	0	0.44	0.44	0.65
Multiple	22	0	0.71	3	0.53	0	0.29	13	0.73	6	0.81	0	0.29	0.74	0.65
Basilar top	7	0	0.29	0	0.82	0	0.11	5	0.91	2	0.69	0	0.11	0.69	0.81
Size of Aneurysm															
Small (<15 mm)	159	1	0.70	14	0.95	1	0.87	109	0.89	34	0.83	0	0.89	0.81	0.81
Large (15-25 mm)	37	1	0.50	3	0.86	0	0.45	22	0.68	11	0.46	0	0.45	0.67	0.39
Giant (>25 mm)	4	0	0.17	1	037	0	0.06	3	0.88	0	0.95	0	0.06	0.50	0.61
WFNS Grade															
Grade I	91	1	1	10	0.62	1	0.73	57	0.73	22	0.80	0	0.73	0.49	0.64
Grade II	42	0	0.99	6	0.35	0	0.42	30	0.80	5	0.20	0	0.42	0.68	0.16
Grade III	51	1	0.56	0	0.11	0	0.48	36	0.83	13	0.77	0	0.48	0.14	0.77
Grade IV	16	0	0.57	2	0.67	0	0.16	11	0.94	5	0.54	0	0.16	0.99	0.67
Male	77	1	0.50	10	0.37	1	0.62	51	0.95	14	0.52	0	0.62	0.27	0.62
Female	123	1	0.85	8	0.46	0	0.70	83	0.96	31	0.66	0	0.81	0.35	0.76
Hypertension (+)	109	1	0.94	9	0.83	0	0.75	74	0.94	25	0.94	0	0.75	0.71	0.96
Diabetes mellitus (+)	71	0	0.82	5	0.63	1	0.46	51	0.74	14	0.69	0	0.55	0.30	0.57
Alcohol (+)	57	1	0.64	10	0.11	1	0.37	33	0.54	12	0.85	0	0.55	0.04	0.85
Smoking (+)	58	0	0.80	8	0.34	1	0.38	38	0.92	11	0.64	0	0.52	0.44	0.78

## Discussion

Subarachnoid hemorrhage caused by the rupture of an aneurysm is a condition with a low chance of recovery and a strong chance of life-long locomotor disability [23]. Guiding physicians in predicting the occurrence and rupture of an aneurysm will help them to save patients’ lives and their capability to be productive. Various genetic polymorphisms are associated with the risk of developing aSAH. Apolipoprotein E polymorphism has emerged as one of the major genetic factors associated with the risk and prognosis of many neurological disorders and of hemorrhagic stroke in various populations.

Japan has the highest incidence rate of aSAH in the Asian population. One explanation for this higher incidence is the relatively high life expectancy in Japan [24]. Statistical reports state that the Japanese population has the highest median age (43 years old) [25].The reported incidence rate of aneurysm in India ranges from 0.75–10.3%, and the North West Indian population, (lowest median age-28 years old) only has an incidence rate of 1% [26]. Therefore, age can be considered a risk factor for aSAH.

Another independent risk factor for aSAH is being female [23]. In our study, incidence of aSAH was 1.6 times higher in the female population than in the male population. The mean age of the female and male population was 55and 40. The reason for the higher incidence rates in women are not still clear. Previously, it was observed that incident rate of aSAH was higher after menopause [27]. After menopause, a drop in sex hormones occurs [28], especially in estrogen. It has also been reported that estrogen has a protective role for SAH [29]. Estrogen has been reported to improve the lipid profile, reducing the risk of atherosclerosis [30, 31], which has been considered an important pathogenic mechanism for the formation of an aneurysm [32].

De Rooij et al. reported that at a younger age, the incidence of aSAH was higher in men than women [1]. But in our study, male and female subjects of less than 50 years of age were equally affected. There were 62.5% females who had multiple aneurysms in our study, which concurs with previous findings that women are more likely to have multiple aneurysms than men [33].

*APOE* polymorphism has been associated with the risk of developing many central nervous system disorders, like Alzheimer’s disease [34–36], vascular dementia [37], multiple sclerosis [38], cerebral infraction [39] and Parkinson’s disease [40]. Many investigations have reported a positive association of *APOE* polymorphism with a risk of aSAH in different populations.

Liu et al. showed that ε2/ε2 and ε2/ε3 genotype frequencies were higher in patients with intracranial aneurysm in the Chinese population [41]. Tang et al. noted that ε4 allele carriers have unfavorable outcomes after aSAH in the Chinese population [42].

In the Japanese population, Kokubo et al. found that ε4 allele carriers have a 2.5-fold higher risk of aSAH [43]. Mineharu et al. suggested that the three alleles of *APOE* did not have any association with aSAH in Western Japan [44].

In the Caucasian population, McCarron et al. reported that the ε2 allele was significantly associated with the risk of different intracranial hemorrhage [13]. Kaushal et al. and Fontanella et al. found that ε2 and ε4 allele were not significantly associated with the risk of aSAH in United States and Italian population [45, 46].

A meta-analysis of nine case control studies found that in the Asian population, ε2/ε2 vs. ε3/ε3, ε2/ε3 vs. ε3/ε3, ε2 vs. ε3 and ε2 allele frequency were associated with the risk of aSAH. However, the study concluded that only the ε2/ε2 vs. ε3/ε3 genetic model was associated with the risk of aSAH in the Caucasian population [21].

In terms of lipoprotein metabolism, the main difference between *APO*ε3 and *APO*ε4 isoforms was that *APO*ε4 has greater affinity for very low-density lipoprotein (VLDL) receptor [47]. Therefore, *APO*ε4 impairs lipolytic processing and leads to the accumulation of VLDL in plasma [48]. The reason for pro-atherogenic lipoprotein–cholesterol distribution in the plasma of the *APO*ε4 isoform was because of the Cys112Arg substitution [49]. The presence of Arg112 in the APOε4 isoform led to an altered interaction between the LDL receptor-binding domain and lipid-binding domain region, which was the reason for the higher binding affinity of this isoform to VLDL [50]. In ischemic stroke patients, the ε3/ε4 genotype was associated with elevated levels of very low-density lipoprotein and triglycerides [22].

Kokubo et al. reported 30% ε3/ε4 genotype frequency among aSAH patients in the Japanese population [43], but in the Chinese population, the ε3/ε4 genotype frequency was found to be 16% [41].

In our study, the ε3/ε4 genotype showed significant association with the risk of aSAH. The frequency for the ε3/ε4 genotype was higher in our patient group (23%) than the controls (14%). We also found that ε4 allele frequency was higher in the patient population, but we did not find any statistical significance.

One of the proposed mechanisms for aneurysm formation and progression is atherosclerosis [51]. It was reported that elevated levels of triglycerides and very low-density lipoprotein promote atherosclerosis [52]. ε4 carriers (ε3/ε4, ε4/ε4, ε2/ε4 genotype) have been associated significantly with risk for atherosclerosis and elevated levels of very low-density lipoprotein [53, 54]. From all these findings, it can be summarized that the ε3/ε4 genotype is one of the risk factor for aSAH, since it can promote atherosclerosis.

In this study, of the aSAH subtypes, the ε2 allele has a significant association with PCOM aneurysm. The PCOM aneurysm is the third most common Circle of Willis aneurysm. PCOM arteries are present at the base of the brain and form a part of the Circle of Willis [55]. It was reported that the ε2 allele is associated with an elevated concentration of plasma triglycerides [56] and that ε2/ε3 genotype carriers were less likely to survive a stroke [57]. Also, the presence of the *APO*ε2 allele may indicate susceptibility to the development of fibrinoid necrosis and microaneurysm [58].

The main difference between APOε3 and APOε2 isoform was that the latter rarely binds to low-density lipoprotein receptors [59]. APOε2 differs from the APOε3 isoform by Arg to Cys amino acid substitution at position 158, which is close to the LDL receptor-binding domain region [60]. Cys158 disrupts the natural salt bridge between Asp152 and Arg154 in the LDLR recognition site, which impairs the binding to the LDL receptor [61]. This makes the APOε2 isoform unable to promote the clearance of low density lipoprotein and triglycerides from the plasma [62]. Bolger et al. noted that one of the reasons for saccular aneurysmal disease is the elevated serum level of low-density lipoprotein [63]. This suggests that the ε2 allele has a role in elevating the levels of low-density lipoprotein and thereby can be a causal factor in aSAH.

## Conclusion

*APOE* polymorphism can be associated with the risk of aSAH in the South Indian population. When compared with the site of aneurysm, the ε2 allele was found to be associated with PCOM aneurysm. Further comprehensive studies are required to confirm these findings.

## Abbreviations

ACOM: Anterior communicating artery
*APOE*: Apolipoprotein E
aSAH: Aneurysmal subarachnoid hemorrhage
CI: Confidence interval
ICA: Internal carotid artery
LDLR: Low-density lipoprotein receptor
MCA: Middle cerebral artery
OR: Odds ratio
PCOM: Posterior communicating artery
VLDL: Very low-density lipoprotein
WFNS: World Federation of Neurological Surgeons
χ2 test: Chi square test
